# Luminescence Nanothermometry: Investigating Thermal Memory in UiO-66-NH_2_ Nanocrystals

**DOI:** 10.1021/acsami.4c06217

**Published:** 2024-07-10

**Authors:** Nour Merhi, Abdullah Hakeem, Mohamad Hmadeh, Pierre Karam

**Affiliations:** Chemistry Department, American University of Beirut, P.O. Box 11-0236, Beirut 1107 2020, Lebanon

**Keywords:** luminescent metal–organic framework, sensing, thermal memory, fluorescence, HEPES

## Abstract

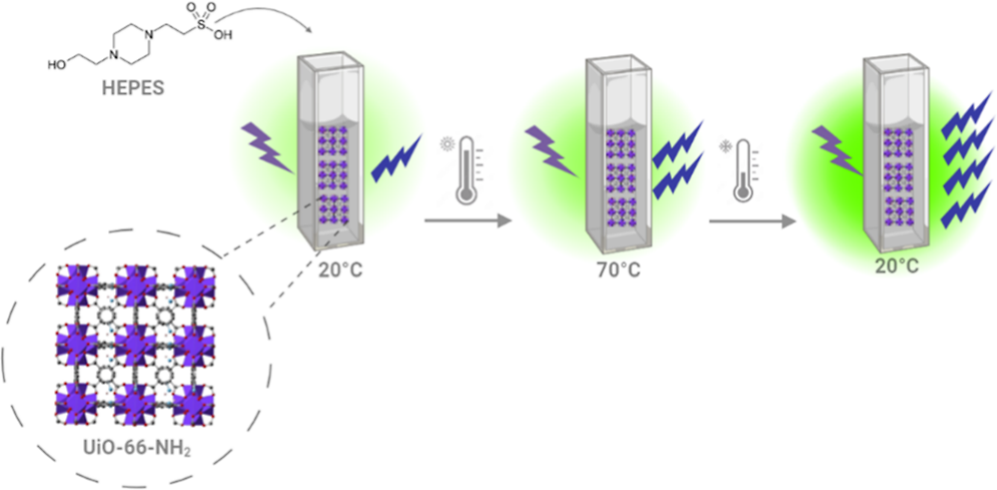

Metal–organic frameworks (MOFs), a diverse and rapidly expanding class of crystalline materials, present many opportunities for various applications. Within this class, the amino-functionalized Zr-MOF, namely, UiO-66-NH_2_, stands out due to its distinctive chemical and physical properties. In this study, we report on the new unique property where UiO-66-NH_2_ nanocrystals exhibited enhanced fluorescence upon heating, which was persistently maintained postcooling. To unravel the mechanism, the changes in the fluorescence signal were monitored by steady-state fluorescence spectroscopy, lifetime measurements, and a fluorescence microscope, which revealed that upon heating, multiple mechanisms could be contributing to the observed enhancement; the MOFs can undergo disaggregation, resulting in a fluorescent enhancement of the colloidally stable MOF nanocrystals and/or surface-induced phenomena that result in further fluorescence enhancement. This observed temperature-dependent photophysical behavior has substantial applications. It not only provides pathways for innovations in thermally modulated photonic applications but also underscores the need for a better understanding of the interactions between MOF crystals and their environments.

## Introduction

Metal–organic frameworks (MOFs), commonly known as MOFs, are tunable coordination networks that are notable for their remarkable features. These include high thermal and chemical stability, crystalline nature, presence of metal nodes, diverse structural dimensions, and porous characteristics.^1,2^ In recent years, MOFs have emerged as modern sensory materials for identifying a wide range of analytes. Owing to their effectiveness, sensors based on MOFs have been proposed to detect a broad spectrum of these substances.^3^ The pore sizes and topologies of MOF structures and their functional groups within the framework’s backbone, such as amino (−NH_2_), thiol (−SH), and hydroxy (−OH), contribute to an enhancement in the MOF’s detection limit.^4,5^ One unique feature of MOFs is that the expansion or functionalization of the organic linkers, while maintaining the identical coordination environment of the inorganic clusters is possible and leads to the formation of MOF structures of the same topology but different pore sizes and pore environments.^6,7^ While the weak intermolecular interactions among the organic linkers certainly contribute to the induction of MOF luminescence, the inorganic and organic entities of the MOF crystals play a more influential role in generating luminescence, either through metal-to-ligand charge transfer or ligand-to-metal charge transfer (LMCT).^8,9^ Therefore, the choice of the organic linker and inorganic cluster appears to be critical in the design of MOF-based sensing materials. Selecting the right material for luminescence-based sensors is essential for the precise detection of target analytes. It is also important for the sensor to remain stable and reusable over time, without losing its effectiveness. The crystalline, tunable, and porous characteristics of MOF structures give them an edge over conventional sensors.^10−12^ This structure not only allows for increased uptake of analytes but also facilitates favorable interactions with the porous and functionalized network, enhancing selectivity by excluding certain species.^13−15^

Temperature is a key physical property in multiple scientific areas, often characterized by its effect on luminescence.^16^ The importance of this factor has been studied for centuries, with its precise measurement and regulation being essential in fields such as manufacturing, energy, and environmental sectors, climate, and health sciences.^17,18^ Generally, measuring temperature involves using a bulky, intrusive probe that physically contacts the object whose temperature needs to be determined (e.g., thermocouples and thermistors).^19^ These probes can be intrusive due to their size, which becomes particularly problematic in micro- and nanoscale systems. The large size of these probes can disrupt processes in micro- and nanoscale systems, making such methods typically ineffective for scales smaller than 10 μm.^20,21^ Recent advancements in thermal sensing, prompted by the limitations of traditional methods, have resulted in the development of contactless thermal sensor detection.^22,23^

Until now, the reported fluorescent metal–organic frameworks are primarily categorized as luminescence derived from organic linkers,^24^ luminescence from lanthanide ions,^25^ luminescence due to charge transfer,^26^ and luminescence induced by guests,^27^ providing a solid theoretical foundation for fluorescence sensing.^28,29^ Among different MOF materials, those based on zirconium clusters show promising potential in fluorescence sensing, attributed to their remarkable chemical stability, diverse structures, and intriguing properties.^30^ It has been reported that lanthanide-based and doped luminescent MOFs (e.g., Eu^3+^/Tb^3+^-MOFs) incorporating linkers of a suitable triplet excited energy state have been recognized as notable temperature sensors.^31−33^ These types of thermometers rely on the ratio of emission intensities between Tb^3+^ (^5^D_4_ → ^7^F_5_) and Eu^3+^ (^5^D_0_ → ^7^F_2_). The emission intensities of these transitions are controlled by the thermally induced energy transfer between the linker and lanthanide metal ions, as well as by back energy transfer. Additionally, phonon-assisted energy transfer from Tb^3+^ to Eu^3+^ contributes to the sensitivity of these two metal ions to temperature, ultimately influencing the emission intensity ratio.^34^ In addition to lanthanide-based MOFs, the creation of a MOF thermometer can also be achieved by introducing fluorophore moieties as guest (e.g., dyes and quantum dots) within the MOF porous network.^35^ UiO-66 derivatives were also doped with lanthanides to develop thin film thermometers by tuning the metal ion composition.^36^ However, to the best of our knowledge, the effect of the temperature on the photophysical properties of Zr-based MOFs, particularly in the context of thermal sensing, has not been previously investigated.

**Scheme 1:**
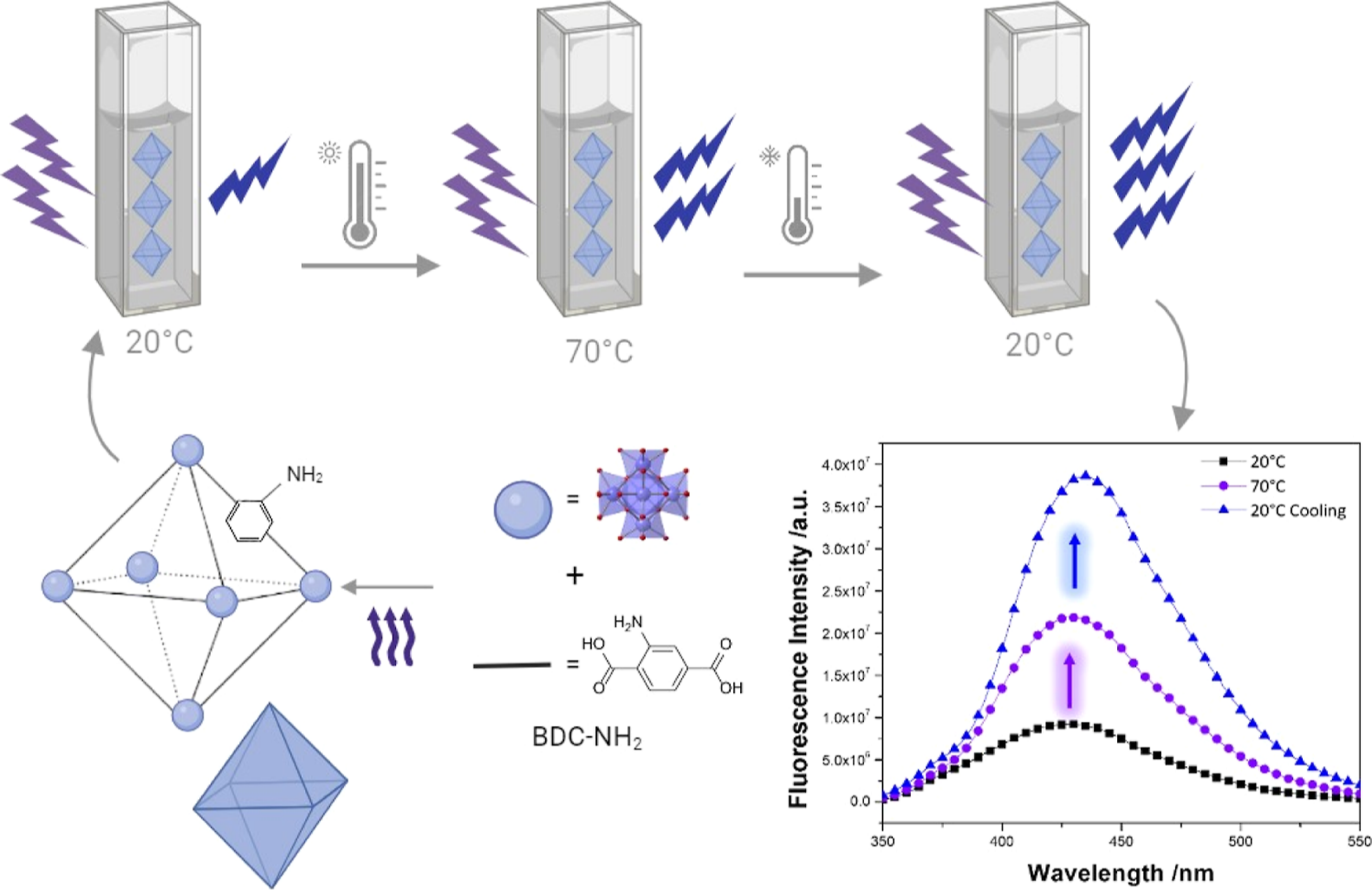
Schematic Representation of the Synthesis, Thermal Cycling, Emission Spectroscopy, and Temperature-Responsive Fluorescence of UiO-66-NH_2_ Suspension The scheme presents the MOF suspension which is fluorescent at room temperature, that is enhanced by two folds upon heating to 70 °C and 4 times after cooling back to room temperature 20 °C demonstrating the concept of “thermal memory”.

In this work, we unveil the unique photophysical properties of UiO-66-NH_2_, a MOF incorporating amine functionality in its backbone that generates a thermal response and memory. Interestingly, UiO-66-NH_2_ showed enhanced fluorescence upon heating, a characteristic that was maintained even after cooling, indicating a distinct thermal memory (Scheme 1). We speculate that the observed fluorescence enhancement is mainly due to the disaggregation and stabilization of colloidal MOF nanocrystals and/or surface-induced phenomena. These findings were characterized and validated under different experimental conditions through various spectroscopic methods, including the use of steady-state fluorescence spectroscopy and a fluorescent microscope, providing a comprehensive analysis of the MOF’s unique thermal response. This observation stands out significantly from traditional MOF-based thermometers that are mainly based on lanthanides or composite materials, including guest species. By leveraging the unique properties of UiO-66-NH_2_ nanocrystals in solution, we achieve a more straightforward and sensitive mechanism for temperature detection.

## Experimental Section

### Materials and General Synthesis Method

Zirconium chloride (ZrCl_4_, 98% purity), formic acid (CH_2_O_2_, 99% purity), 2-aminoterephthalic acid (C_8_H_7_NO_4_, 99% purity), and 4-(2-hydroxyethyl)-1-piperazineethanesulfonic acid (1 M HEPES) were supplied by Sigma-Aldrich. Additionally, organic solvents of high purity (99%) were purchased from Fisher Scientific. In all experiments, deionized water with a resistivity of 18.2 MΩ·cm was utilized. Thermal gravimetric analysis (TGA) was conducted by using the NETZSCH TG209 F1 Libra instrument. Powder X-ray diffraction (PXRD) patterns were recorded by a Bruker D8 ADVANCE X-ray diffractometer at 40 kV and 40 mA (1600 W), using Cu Kα radiation (*K* = 1.5418 Å). Scanning electron microscopy (SEM) analysis was performed with a MIRA3 Tescan electron microscope, precoating the samples with a fine gold layer. The Brunauer–Emmett–Teller (BET) surface area measurements were made by using a Quantachrome-NOVA 2200e surface area and pore size analyzer. UV–visible–NIR absorption spectra were recorded at ambient temperature on a JASCO V-570 spectrophotometer. Fluorescence measurements were carried out using a HORIBA Jobin Yvon Fluorolog-3 equipped with a temperature controller unit (T3 Quantum Northwest). The excitation source was a 100 W xenon lamp, and the R-928 detector was operated at 950 V.

UiO-66-NH_2_ was synthesized solvothermally following a well-established procedure using formic acid as modulator.^37^ Briefly, 800 mg of ZrCl_4_ (3.4 mmol) and 566 mg of aminoterephthalic acid (3.4 mmol) were dissolved in 250 mL of dimethylformamide (DMF) by sonication. After complete dissolution, 11 mL of formic acid was added to the mixture and the obtained homogeneous solution was placed in a preheated oven at 120 °C for 24 h. The obtained yellow precipitate was then transferred to a Falcon tube for centrifugation to collect the MOF crystals which were washed three consecutive times with DMF and dichloromethane, respectively. Finally, UiO-66-NH_2_ crystals were dried in a vacuum oven at 150 °C overnight for thermal activation.

For fluorescence measurements, a MOF solution of 0.125 mg/mL was prepared in HEPES buffer (10 mM) containing 150 mM NaCl (pH 7.3) unless otherwise stated. Emission spectra and time scans were recorded following excitation at 330 nm. To ensure a uniform temperature distribution, the prepared sample was continuously stirred at 400 rpm during the analysis. Typically, for fluorescence experiments, the sample was initially set in the fluorometer at 20 °C and allowed to stabilize at 20 °C for 2 min. The temperature was then raised to 70 °C and sustained for 10 min. Subsequently, the temperature was gradually reduced to 20 °C.

For the single particle imaging, 20 μL of a UiO-66-NH_2_ solution was added on a glass slide, which was then covered with a coverslip. The slide was placed on a LINKAM PE120, a Peltier-controlled heating stage equipped with a Leica DM6 B fully automated upright fluorescence microscope (excitation 325–375 nm and emission 435–485 nm) and a 10× objective lens. The heating stage allowed for the temperature of the slide to be adjusted and maintained; the slide was heated to 70 °C and subsequently cooled to 20 °C, and images were captured at each temperature. Temperature regulation is controlled by the Linkam T95 controller, which links the PE120 thermal stage to a computer using specialized software.

## Results and Discussion

**Figure 1:**
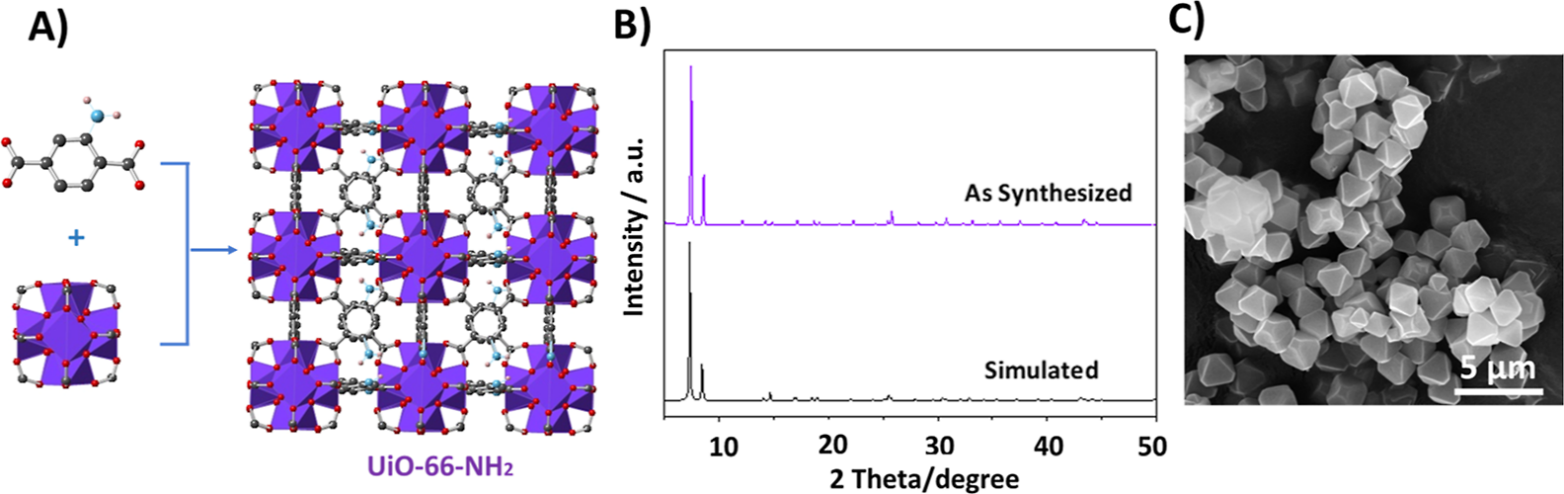
(A) Crystal structure of UiO-66-NH_2_, (B) PXRD pattern of the as-synthesized UiO-66-NH_2_ compared to the simulated one, and (C) SEM image of the obtained UiO-66-NH_2_ crystals.

UiO-66-NH_2_ crystals were synthesized following previously published procedures.^37,38^ The crystalline nature and phase purity of the sample were verified through PXRD analysis, as depicted in Figure 1 which shows that the PXRD pattern of the as-synthesized UiO-NH_2_ perfectly matches the simulated one. The SEM image of the obtained crystals also demonstrated that the UiO-66-NH_2_ sample was pure, featuring uniformly shaped truncated octahedral crystals, each approximately 200 nm in size. Such a crystal configuration is characteristic of UiO-based MOF structures.^39,40^

To evaluate the porosity of the UiO-66-NH_2_ framework, the nitrogen adsorption/desorption isotherm was measured for the activated sample (Figure S1A). Subsequently, applying the BET technique revealed a surface area of 1700 m^2^/g, aligning with previously published values.^37^ TGA was performed to determine the thermal stability and to estimate the defect number in the MOF nanocrystals. UiO-66-NH_2_ was shown to degrade at a temperature of 350 °C under air and the defect number was calculated based on the TGA to be 2.0 missing linkers per cluster (Figure S1B).

### Thermal Memory Experiments

Fluorescence and absorbance spectra of UiO-66-NH_2_ and its free linker are first recorded at 20 °C and presented in Figure S2. The absorbance band at 370 nm of the MOF is attributed to LMCT. This absorption band is the result of an *n*–π* transition of the aminoterephthalate linker which is followed by electron injection from a π* orbital to the zirconium cluster.^41^ The main broad fluorescence peak of the MOF is centered at 440 nm, which is slightly red-shifted when compared to the aminoterephthalic acid linker. It has been noticed that the emission behavior of the linker within an MOF differs from its free state in terms of both peak position and intensity. This shift could be attributed to the coordination between the metal ion and the organic linker.^42^

**Figure 2:**
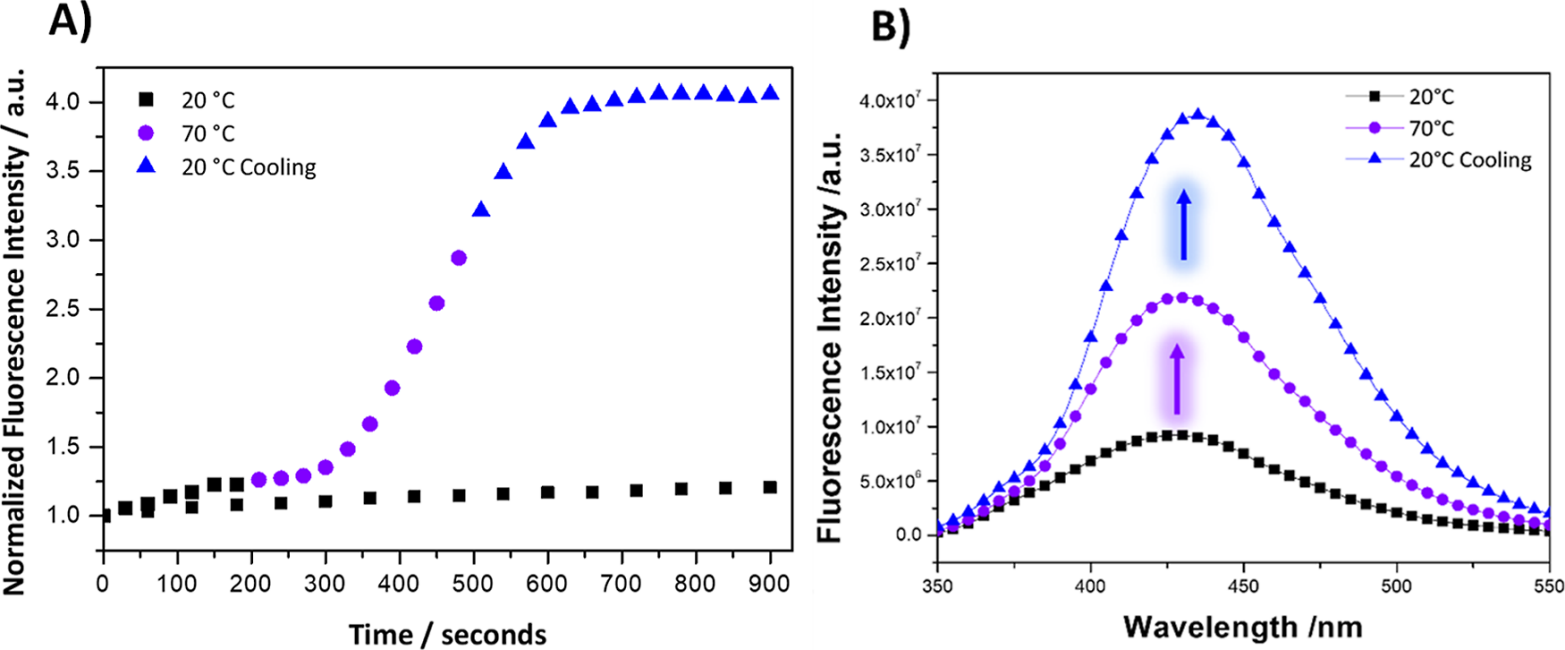
(A) Fluorescence intensity time trajectory of 0.125 mg/mL UiO-66-NH_2_ overtime. The time trajectory was recorded at 450 nm upon excitation at 330 nm. (B) Fluorescence emission of 0.125 mg/mL UiO-66-NH_2_ recorded at an excitation of 330 nm and emission of 350–550 nm. Temperature profile (Δ) of the solution upon changing the temperature from 20 to 70 °C and back to 20 °C as recorded by the external thermocouple. All experiments were carried out in buffer solutions containing 150 mM NaCl and 10 mM HEPES, with a pH level maintained at 7.3.

The fluorescence emission spectra of UiO-66-NH_2_ in 10 mM HEPES buffer and 150 mM of NaCl (pH = 7.3) were recorded under different temperatures (Figure 2B). At room temperature, the sample exhibits a fluorescence band centered at 440 nm, which is attributed to the aminoterephthalate linker. When heated to 70 °C, a 2-fold fluorescent enhancement is observed. Remarkably, as the sample cools back to room temperature, the fluorescence intensity amplifies further to approximately 4-fold the initial room temperature intensity. This intensity transition was clearly observed when the fluorescence intensity was recorded as a function of time over 900 s. Indeed, the fluorescence signal increases as soon as the solution starts to heat up, and it continues to do so even after cooling while reaching a constant value after a few seconds. It is noteworthy to mention that the fluorescence intensity maintained its initial value when the temperature was not changed (20 °C) over the same period of time.

Interestingly, the free linker (Amino-BDC) did not show a similar response when subjected to the same temperature changes; in contrast, a slight decrease in the fluorescence intensity was observed upon heating, which is consistent with the increase in the nonradiative pathway upon temperature increase (Figure S3).

**Figure 3:**
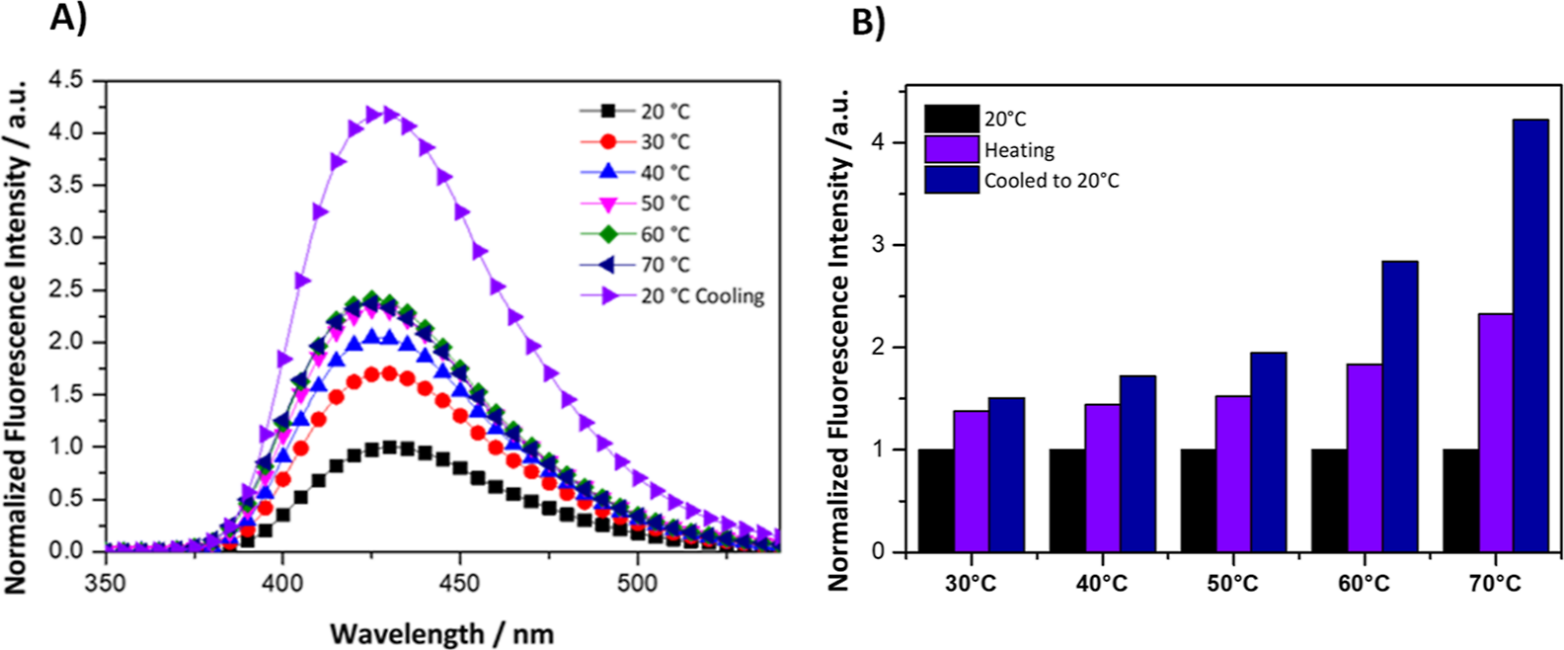
(A) Fluorescence intensity emission spectra of a 0.125 mg/mL UiO-66-NH_2_ in 10 mM HEPES with 150 mM NaCl with incremental temperature elevations of 10 °C from 20 to 70 °C followed by cooling back to the ambient temperature 20 °C and (B) normalized fluorescence intensity of samples at a temperature of 20 °C, during a heating cycle across a temperature range from 30 to 70 °C, and after the sample has been cooled back to 20 °C.

The experimental results shown in Figure 3 consisted of a 10 °C incremental increase of the solution temperature, starting from an ambient temperature of 20 °C and progressing through to 70 °C and subsequently upon cooling to the initial temperature 20 °C. The fluorescence intensity of the MOF solution was measured at each temperature interval. Observations indicate a general trend of increasing fluorescence intensity with rising temperature. This observation reflects the temperature-induced enhancement of the fluorescent properties of the MOF. Upon cooling, the fluorescence intensity does not revert to the initial value at 20 °C but instead remains at an elevated level. Furthermore, the fluorescence intensity was measured for five samples upon exposing each to different temperatures ranging from 30 to 70 °C and subsequently cooled back down to 20 °C (Figure 3B). During the heating process, the fluorescence intensity increases with temperature, peaking notably for the sample heated to 70 °C. Once cooled to 20 °C, the intensity enhances more and remains elevated compared to the initial state, indicating also a “thermal memory”.

### Stability of UiO-66-NH_2_ after Heating–Cooling Cycles

We speculated that the observed fluorescence intensity enhancement could be the result of multiple phenomena: disaggregation of the MOF particles, leaching of the linker, or other surface-induced mechanisms. As such, we designed several experiments to pinpoint the most probable mechanism/s.

Upon heating the MOF sample, a change in the turbidity of the suspension was observed. Initially, the suspension in the “Before Heating” vial shows turbidity, suggesting a higher degree of particle agglomeration or larger particle size. Conversely, the “After Heating” vial displays a significant reduction in turbidity (Figure S4). This observation is also supported by dynamic light scattering (DLS) where a decrease in the hydrodynamic radius of the MOF suspension was observed from an average of 4000 nm before heating down to 1500 nm after heating (Figure S5). The clarity observed postheating can be a result of enhanced particle suspension and thermal disaggregation, leading to more colloidally stable MOF nanocrystals.

**Figure 4:**
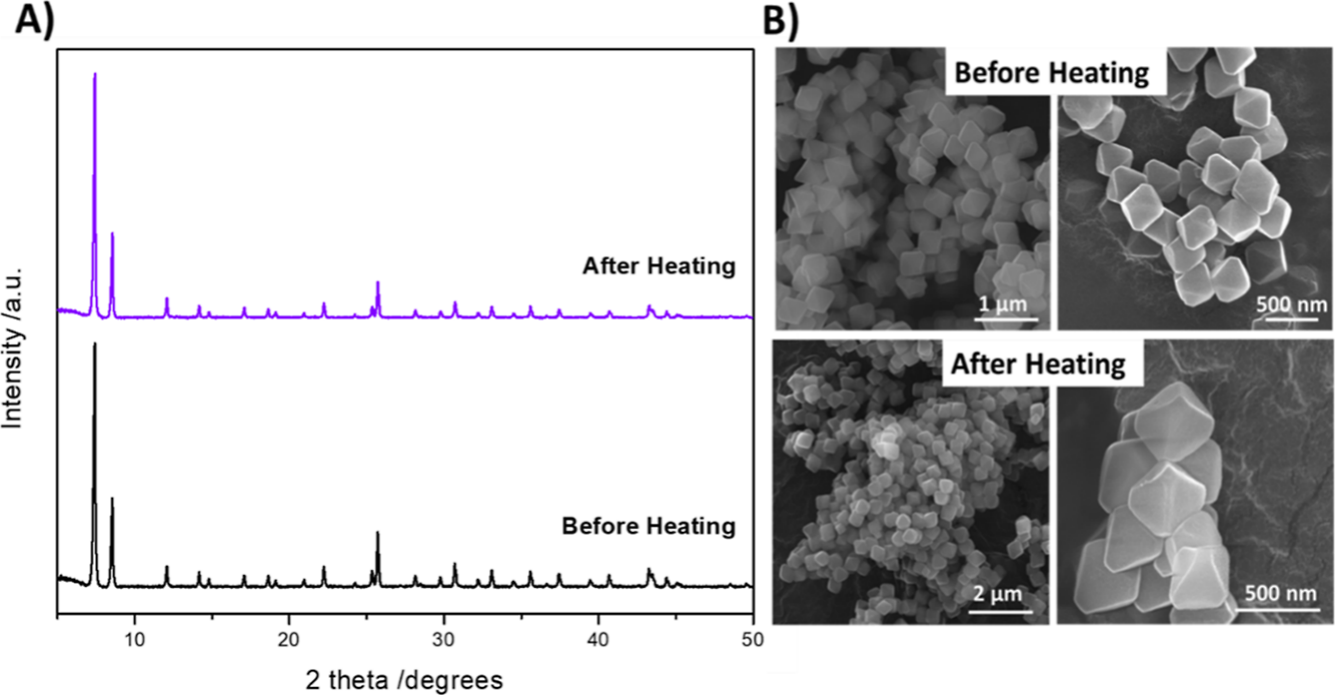
(A) PXRD patterns and (B) SEM images of the UiO-66-NH_2_ nanocrystals before and after heating.

To rule out the possibility of the linker leaching through any potential thermally induced destabilization of the MOF under our experimental conditions, SEM, PXRD, AA, and lifetime measurements are investigated at 70 °C. PXRD and SEM show that there is no change in the crystallinity of the sample after heating (Figure 4). As for the AA analysis of the supernatant, no Zr metals are detected, which demonstrated the stability of the UiO-66-NH_2_ crystals under our experimental conditions.

The fluorescence lifetime is an important photophysical parameter that can provide insights into the environmental dynamics of the excited state of a molecule. As such, it can potentially distinguish between the free linker and the framework-bound linker. The fluorescence lifetimes of UiO-66-NH_2_ and the 2-aminoterephthalate linker were measured in different media and ionic strengths, before and after heating the samples. A summary of these measurements is reported in Table 1 and Table S1. The linker lifetime was measured to be equal to 16.2 ns with no noticeable changes when it was heated to 70 °C for 10 min. When locked into the MOF structure, the linker exhibited a decrease in its lifetime, indicating a quenching mechanism in place. To address whether there is leaching of the linker upon heating, we conducted a series of additional lifetime measurement experiments; if the linker was leaching into the solution, we should detect a subpopulation of a lifetime that matches that of the free linker. As such, a MOF solution was heated for 10 min at 70 °C then cooled to room temperature before measuring its lifetime. The recorded lifetime was again fitted into a monoexponential with no change in the measured lifetime. We also centrifuged down the large MOF particle at 6000 rpm for 5 min since we assumed any leached linker would remain in the supernatant. The lifetime measurement of the supernatant matched that measured for the MOF. This suggests that the species responsible for fluorescence in the supernatant are most probably colloidally stable small particles of the MOF rather than dissociated linker molecules, which is in line with the observed reduced aggregation after heating. The lack of significant variation between the lifetimes before and after heating reinforces the theory that no substantial linker is leaching out of the MOF structure upon thermal treatment. While all of the evidence points out that the observed fluorescence enhancement is not due to a linker leaching out of the solution, we cannot rule out this mechanism entirely. Bůžek et al. studied the stability of UiO-66 in different buffers.^43^ It was found that HEPES buffers were the most compatible, causing only 9% degradation of the MOF after 3 h of incubation. In contrast, Tris buffers, phosphate buffer, and *N*-ethylmorpholine solutions led to more significant degradation, with complete dissolution of the MOF at higher concentrations or shorter exposure times.^43^

**Table 1:** Fluorescence Lifetime Data of UiO-66-NH_2_, Its Linker, and the Supernatant of the System in Different Media and Ionic Strengths before and after Heating

	lifetime (ns)	
sample	before heating	after heating
2-aminoterephtalate linker in HEPES	16.2	16.3
UiO-66-NH_2_ in deionized water	highly aggregated (no signal)	15.7
UiO-66-NH_2_ in 10 mM HEPES + 150 mM NaCl	15.5	15.8
UiO-66-NH_2_ in 10 mM HEPES + 150 mM NaCl supernatant	15.5	15.4

### Fluorescence Microscopy Imaging

**Figure 5:**
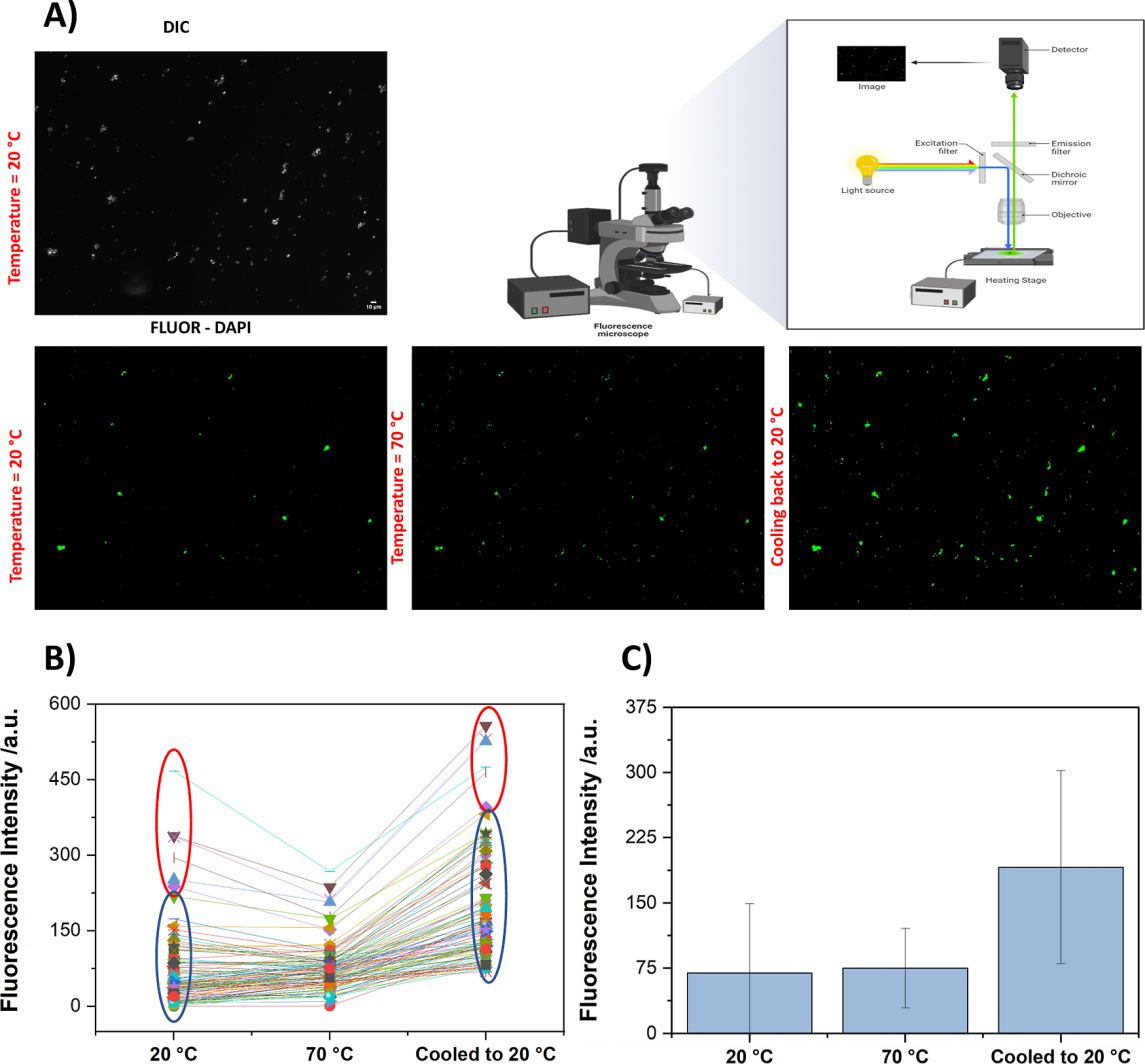
(A) Thermal modulation of UiO-66-NH_2_ particles via fluorescence microscopy, where the DIC micrograph is shown on the top, and the fluorescent micrographs of the particles throughout the heating cycle are shown at the bottom. Fluorescence Intensity of UiO-66-NH_2_ particles (B) and their average intensity (C) tracked before (20 °C) and after heating (70 °C) and upon cooling (20 °C). The red (representing large particles) and blue (representing small particles) circles designate the two subpopulations present in the sample.

We next tracked the fluorescence changes of single UiO-66-NH_2_ particles with temperatures. Fluorescence images were acquired at 20 °C and then using a heating stage, the sample temperature was increased to 70 °C. A side-by-side comparison at ambient and elevated temperatures, along with a schematic of the experimental setup, provides a comprehensive overview of the methodological approach and the resultant effects on particle behavior (Figure 5).

Fluorescence intensity from individual particles was measured and tracked as the temperature was increased and then cooled back to 20 °C. Upon heating, a marked increase in fluorescence intensity of the particles, suggesting a thermally induced enhancement of the photophysical properties, is observed. After cooling the suspension back to 20 °C, the fluorescence does not revert back to its initial state; instead, the particles maintain an elevated fluorescence, implying irreversible changes induced by the thermal cycle. When changes in individual particles are examined, we observed a trend where UiO-66-NH_2_ particles with lower initial fluorescence intensities at 20 °C exhibit a greater percentage of fluorescence enhancement upon heating. This inverse relationship suggests that smaller or less fluorescent particles may be more responsive to thermal activation, potentially due to a larger surface-to-volume ratio, which facilitates more effective heat-induced photophysical changes. This is probably due to their increased access to adsorption sites when normalized to the total volume. Moreover, the fact that we observed fluorescence enhancement from individual particles underscores the fact that the enhancement could not be primarily due to the linker leaching out of the MOF particles since such an event will not be detected by fluorescent microscopy images as small molecules diffuse fast out of the focal plane.

The bar graph in Figure 5C displays the fluorescence intensity data, showing the mean response of the particles at 20 and 70 °C and after cooling back to 20 °C. The mean fluorescence intensity at 70 °C is higher than at 20 °C, which is enhanced and retained compared to the initial intensity, indicating that the thermal enhancement effect is more prominent in particles with initially lower fluorescence. These observations collectively point to size-dependent thermal responsiveness in UiO-66-NH_2_ particles. To further confirm this hypothesis, we tested the fluorescence emission of MOF particles at the same concentration upon sonication at different time intervals. The obtained emission spectra are shown in Figure S6 and reveal a clear trend of increasing signal with longer sonication times, thus confirming that the disaggregation of the particles leads to fluorescence enhancement.

**Figure 6:**
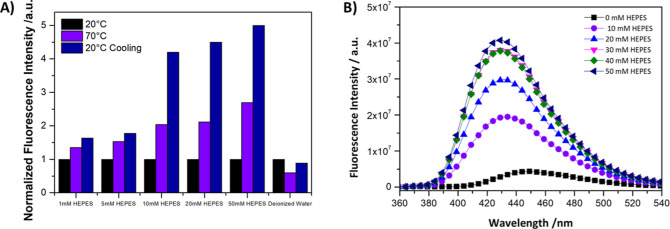
(A) Quantitative analysis of fluorescence intensity increase in a MOF system with progressive HEPES buffer concentrations and (B) comparative fluorescence emission spectra of a MOF in deionized water upon titration with different HEPES concentrations.

Sulfonates moieties have been reported to bind to the exposed Zr-sites in UiO-66 as evidenced by the high adsorption capacities of UiO-66 and its amino-functionalized version toward perfluorooctanesulfonate (PFOS) and their binding increases with the increase in temperature.^44,45^ Indeed, it has been demonstrated that anion adsorption on the defect sites of the UiO-66 defected frameworks is an endothermic process by which the affinity of the anions (e.g., arsenates, phosphates, and sulfonates) to the MOF increases with temperature.^46^ The high fluorescence intensity retained upon cooling is probably due to the irreversible nature of the binding of HEPES’s sulfonates to the defect sites on the Zr-cluster of the UiO-66-NH_2_. It is noteworthy that two missing linkers per Zr-cluster were calculated for the UiO-66-NH_2_ which could explain a potential binding of HEPES to the defected clusters within UiO-66-NH_2_ nanocrystals. In this work, HEPES was initially used for its buffer capacity properties, but it has a sulfonate moiety, which can potentially bind to the exposed Zr-sites in UiO-66-NH_2_ nanocrystals. To elucidate any effect that the HEPES might have on the fluorescence intensity of UiO-66-NH_2_, incremental amounts of the HEPES were added. A steady increase in the fluorescence signal was observed (Figure 6). To rule out the effect of the change in the ionic strength, incremental amounts of NaCl were added with no observed changes (Figure S7).

Additionally, the time scan of the MOF in deionized water showed no response, further indicating that the observed thermal response is specific to the MOF’s assembly environment (Figure S8). This observation was also reported by Wang and Wang who observed no changes in the fluorescent signal of UiO-66-NH_2_ upon heating in pure water in the absence of HEPES.^47^

As depicted in Figure 6A, the fluorescence intensity of UiO-66-NH_2_ was measured in matrices of 1 mM, 5 mM, 10 mM, and 50 mM HEPES buffers all with 150 mM NaCl, as well as in deionized water. These measurements were taken at ambient temperature (20 °C), after heating to 70 °C, and following a cooling period back to 20 °C. The data indicate a pronounced increase in fluorescence intensity upon heating to 70 °C across all HEPES buffer concentrations. The fluorescence intensity in samples with higher concentrations of HEPES buffer postcooling exceeded the initial ambient temperature values. This intriguing behavior points to a potential thermally induced restructuring within the MOF that stabilizes in a configuration with an enhanced fluorescence emission. The fluorescence emission spectra shown in Figure 6B represent the titration of the UiO-66-NH_2_ in deionized water with varying concentrations of HEPES buffer. Each curve corresponds to a different concentration, with the fluorescence intensity enhanced as a function of increasing HEPES concentration. These data highlight the importance of HEPES in this phenomenon. This is in agreement with the previously reported study highlighting that sulfonate-based functional groups have a great affinity to the defected sites on the Zr-MOF clusters as evidenced by the high adsorption capacities of PFOS and perfluorobutanesulfonate by defected UiO-66 and UiO-66-NH_2_ adsorbents.^44,45^ To better understand the role of defects in retained fluorescence enhancement, we synthesized a new batch of UiO-66-NH_2_ without using a modulator. PXRD, SEM, and TGA of the obtained MOF were recorded and reported in Figure S9. The thermal response is then tested under the same experimental conditions as the modulated version, and it is found that, while it exhibits a fluorescence enhancement upon heating and cooling, it is not as pronounced as that in the defected version (Figure S9). This observation supports the idea that defects may influence the observed fluorescence enhancement. In addition, it shows the importance of defects in enhancing the interactions with HEPES which was found to be critical for the thermal response by forming colloidally stable MOF particles. It is noteworthy to mention that the nondefected particles are of relatively smaller size (100 nm) which resulted in better suspension in solution, and therefore a higher initial fluorescence intensity was obtained.

The zeta potential measurement of the suspended nanocrystals at 20 °C and upon heating to 70 °C shows a change in the surface charge from +0.7 to −2.3 upon heating which was retained negative upon cooling (Figure S10). This is probably due to the strong interactions of the sulfonate moieties of the HEPES with the surface of the MOF nanocrystals.

In deionized water, the fluorescence intensity remained minimal across all temperatures tested, implying an environmental dependency on the photoluminescence of UiO-66-NH_2_. The absence of necessary ionic components could explain the suppressed fluorescence in this matrix.

To gain deeper insights into the temperature responsiveness and determine if the enhanced emission establishes a new starting point, we conducted further thermal cycling experiments following the initial observed enhancement (Figure S11). After the first cycle, the fluorescence intensity decreases with the increase in temperature but then increases upon cooling, surpassing the initial intensity; this pattern repeats again in the following cycle. This behavior indicates the presence of multiple mechanisms: all speculated mechanisms are active in the first cycle, but from the second cycle onward, the decrease in the fluorescence intensity upon heating suggests that at least one mechanism responsible for the initial enhancement is suppressed.

## Conclusions

In conclusion, in this work, a novel thermally induced fluorescence response in the amino-functionalized Zr-MOF UiO-66-NH_2_ is reported. The distinctive photophysical memory exhibited by this MOF is characterized by enhanced fluorescence upon heating. The observed behavior, which could be driven by disaggregation and/or leaching of linkers and/or interactions with HEPES, to form colloidally stable UiO-66-NH_2_ nanocrystals underscores the importance of understanding the dynamics between defect-rich MOFs and their environment. It is important to note that our study primarily emphasized on the behavior of smaller MOF particles, rather than the larger, possibly agglomerated ones since they exhibited a more pronounced fluorescence response upon heating, indicating that size plays a vital role in the photophysical properties of these materials. The ability to modulate fluorescence thermally opens up new possibilities for the design of smart materials and devices that can respond to temperature changes. Moreover, this work lays the groundwork for future investigations into better understanding the underlying mechanisms of thermally induced fluorescence in MOFs and other crystalline materials.

## Supporting Information Available

Experiments such as nitrogen adsorption–desorption isotherm, TGA, defect calculation of UiO-66-NH_2_ nanocrystals, UV–vis, control fluorescence measurements, DLS analysis, characterization of the UiO-66-NH_2_ obtained via nonmodulated synthesis, fluorescence lifetime measurements, and zeta potential measurements (PDF)
