# Supplementary material for: Luminescence Nanothermometry: Investigating Thermal Memory in UiO-66-NH2 Nanocrystals
Source: ACS Appl Mater Interfaces. 2024 Jul 10;16(29):38702–10. doi: 10.1021/acsami.4c06217 (PMC11284752; doi:10.1021/acsami.4c06217)
Supplement: Supplementary file 1 — am4c06217_si_001.pdf [file am4c06217_si_001.pdf]

SUPPORTING INFORMATION

Luminescence Nano-Thermometry: Investigating Thermal Memory in UiO-66-NH_2 Nanocrystals

*Nour Merhi, Abdullah Hakeem, Mohamad Hmadeh *, and Pierre Karam**

Chemistry Department, American University of Beirut, P.O.Box 11-0236, Riad El-Solh, 1107 2020 Beirut, Lebanon

*Mohamad Hmadeh: mohamad.hmadeh@aub.edu.lb *Pierre Karam: pierre.karam@aub.edu.lb

Table of Contents

***Figure S1**: Nitrogen adsorption-desorption isotherm, TGA, and defect calculation of UiO-66-NH_2 nanocrystals.* *S2*

Figure S2: Absorption and Emission Spectra of UiO-66-NH_2 nanocrystals in HEPES with NaCl ***S3***

Figure S3:Thermal response of the linker.. .... ***S4***

Figure S4: Comparison of cuvettes before and after heating ***S4***

Figure S5:Dynamic light scattering analysis ***S5***

Table S1:Fluorescence lifetime measurements of UiO-66-NH_2 and its linker ***S6***

Figure S6:Emission spectra of the MOF upon different sonication time ***S6***

Figure S7:Emission Spectra upon titration with different concentrations of NaCl ***S7***

Figure S8:Intensity time trajectory of the MOF in deionized water ***S7***

Figure S9:Characterization of the UiO-66-NH_2 in the absence of modulator ***S8***

Figure S10:Zeta potential at different temperature ***S9***

Figure S11: Normalized fluorescence intensity at different thermal cycles ***S10***

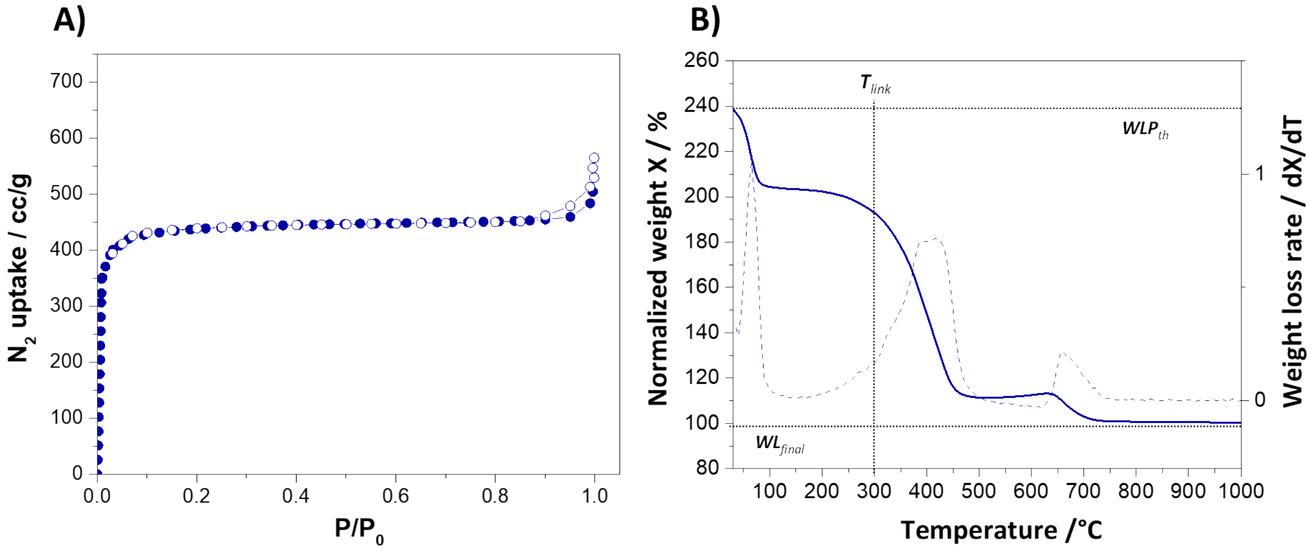

Figure S1: A) Nitrogen adsorption-desorption isotherm recorded at 77k for UiO-66-NH_2. • Adsorption °Desorption and B) Thermogravimetric analysis (TGA) and derivative thermogravimetric (DTG) curves of UiO-66-NH_2. The solid lines on the left axis depict the TGA curves, while the dashed lines on the right axis illustrate the DTG curves. The lower black pointed horizontal line indicates the lower limit of the theoretical TGA weight loss plateau, denoted as WL_{final}. The upper pointed horizontal line signifies the upper limit of the theoretical TGA weight-loss plateau, represented as WLP_{th}. The vertical pointed line marks the temperature at which the linker combusts, labeled as T_{link}.

Defect Calculation:

The defect number in the hydroxylated Zr-MOF, with the general formula $Zr_6O_4(OH)_4(linker)_6$, was determined based on a well-established method from the TGA analysis. Using this calculation, it is assumed that under an air flow, the final product of the framework's combustion is 6 ZrO_2. The TGA curve was normalized so that the final weight WL_{final} is set to 100%. Consequently, the theoretical weight loss WLP_{th} can be calculated as follows:

$$WLP_{th} = \frac{MW_{MOF}}{MW_{6ZrO_2}} \times WL_{final}$$

However, the given chemical formula of the MOF describes an ideal structure, where the theoretical number of linkers connected to one Zr6 unit is $NL_{th} = 6$. The weight loss corresponding to a single linker WL_{link} can be determined by dividing the total weight loss by the total number of linkers as follows:

$$WL_{link} = \frac{WLP_{th} - WL_{final}}{NL_{th}}$$

The actual frameworks are rather defective, with the number of linkers NL_{exp} being less than NL_{th}. To estimate this number, it is necessary to identify the temperature beyond which no more desolvation, dehydroxylation, or modulator loss occurs. This temperature, T_{link} is defined as the point at which the MOF structure consists only of the Zr_6 unit connected to NL_{exp} organic linkers. The first derivative of the TGA curve is plotted to better identify the major mass losses for each MOF structure. The normalized weight corresponding to T_{link} is denoted as WLP_{exp}. The weight loss occurring beyond this point is attributed to the combustion of the linkers. Consequently, NL_{exp} is expressed as follows:

$$NL_{exp} = \frac{WLP_{exp} - WL_{final}}{WL_{link}}$$

The number of missing linkers is finally obtained by the following subtraction:

$$NL_{mis} = NL_{th} - NL_{exp}$$

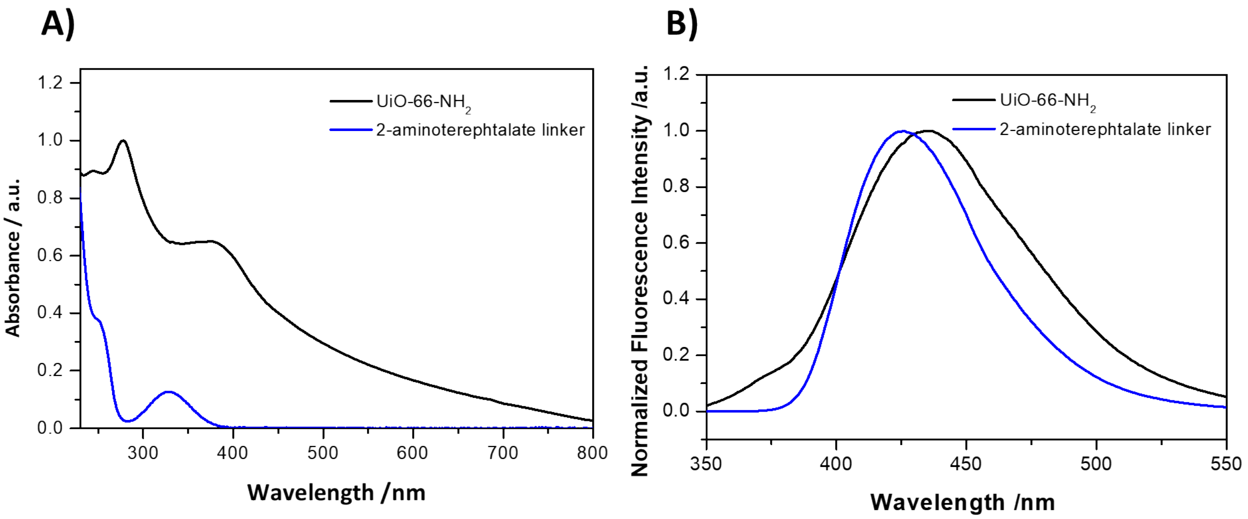

Figure S2: A) UV-Vis absorption spectrum of 2-aminoterephthalate linker in 10 mM HEPES with 150 mM NaCl. The spectrum exhibits an absorption peak at 330 nm, which is utilized for the excitation of our MOF system, and B) Emission spectra of UiO-66-NH_2 and its 2-aminoterephthalate linker in 10 mM HEPES and 150 mM NaCl.

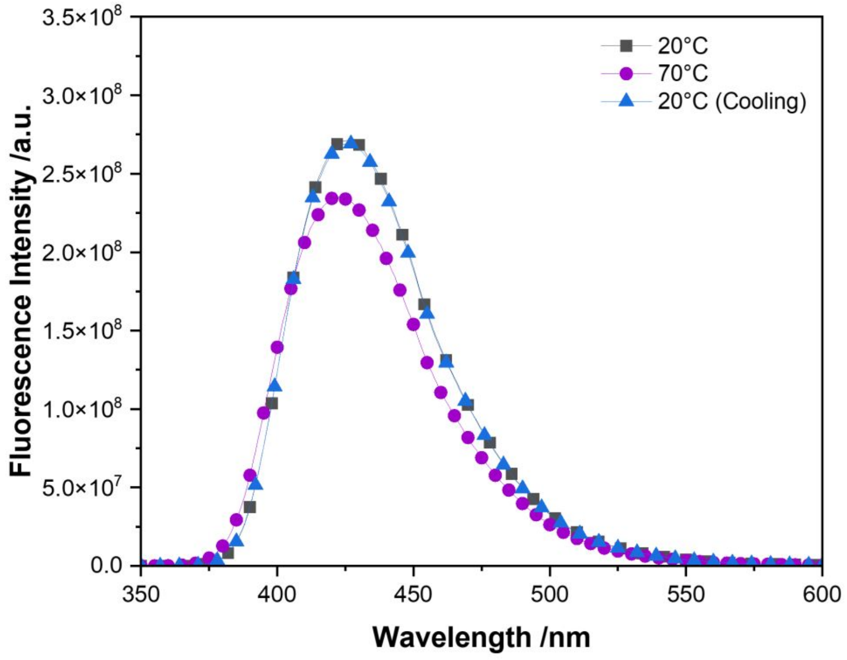

Figure S3: Emission spectra of aminoterephthalate linker in 10 mM HEPES with 150 mM NaCl at different temperatures 20°C, 70°C, and after returning to 20°C following cooling. The overlay of the spectra emphasizes the absence of thermal responsiveness of the linker, with negligible shift in peak wavelength, indicating a stable fluorescent property across the tested temperatures.

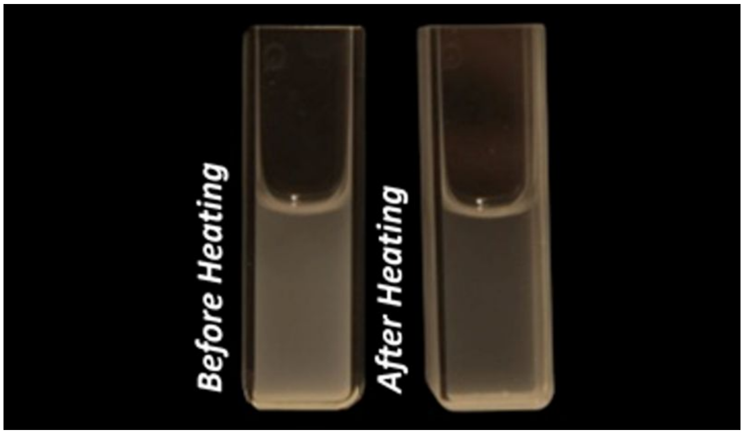

Figure S4: Side-by-side comparison of two vials containing UiO-66-NH2 suspensions in HEPES solution with NaCl before (20 °C) and after heating them to 70 °C.

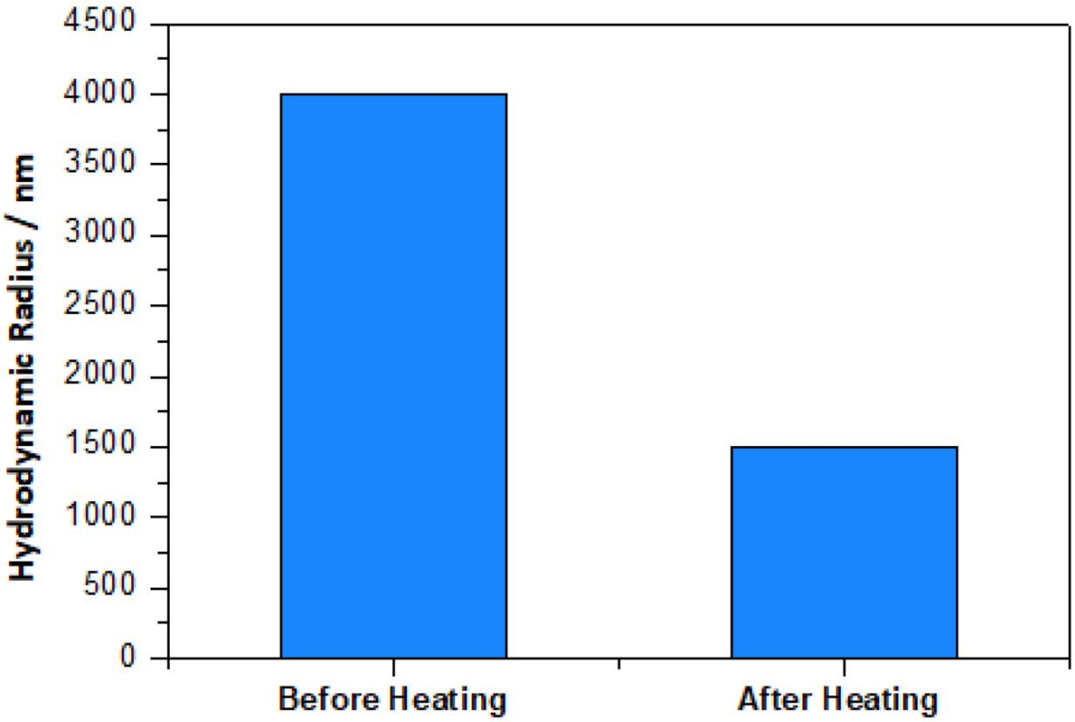

Figure S5: Dynamic Light Scattering (DLS) analysis of UiO-66-NH_2 in 10 mM HEPES with 150 mM NaCl suspension particle size before and after heating. The bar graph depicts a significant reduction in the average particle diameter from approximately 4000 nm before heating to around 2000 nm post-thermal treatment which marks a change in the particle aggregation state due to the heating cycle.

Table S1: Fluorescence Lifetime Measurements of UiO-66-NH_2 and its Linker in Different Media and Ionic Strengths Before and After Heating

Sample	Lifetime (ns)	
	Before Heating	After Heating
**2-aminoterephtalate Linker in HEPES**	16.2	16.3
**UiO-66-NH_2 in Deionized Water**	Not visible	15.7
**UiO-66-NH_2 in 2mM HEPES**	15.4	15.7
**UiO-66-NH_2 in 5mM HEPES**	15.4	15.7
**UiO-66-NH_2 in 10mM HEPES**	15.4	15.7
**UiO-66-NH_2 in 10mM HEPES + 50mM NaCl**	15.4	15.8
**UiO-66-NH_2 in 10mM HEPES + 100mM NaCl**	15.5	15.8
**UiO-66-NH_2 in 10mM HEPES + 150mM NaCl**	15.5	15.8
**UiO-66-NH_2 in 10mM HEPES + 150mM NaCl Supernatant**	15.5	15.4

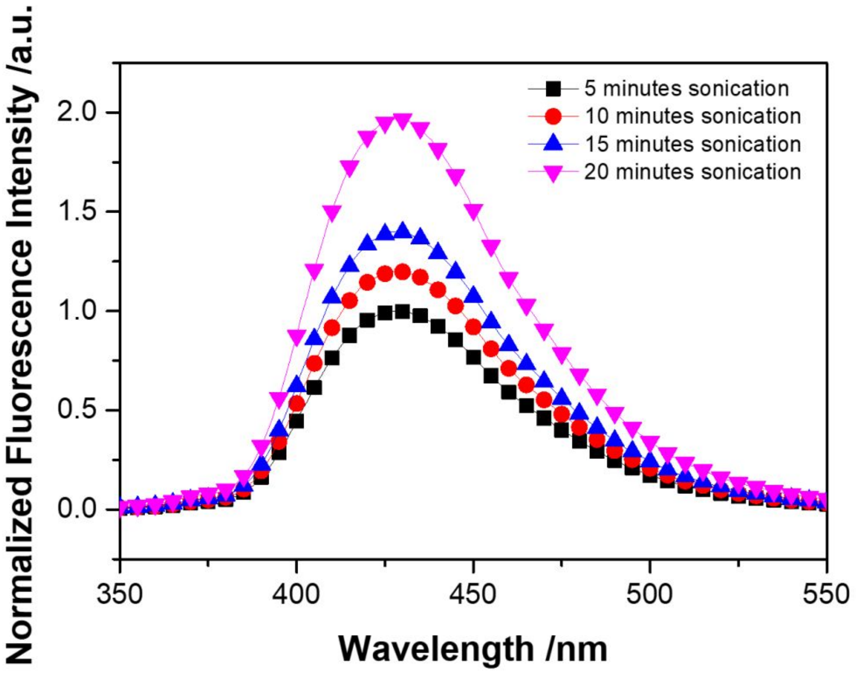

Figure S6: Emission Spectra of 0.125 mg/mL UiO-66-NH2 in 10 mM HEPES with 150 mM NaCl upon sonication at different durations at 20 °C.

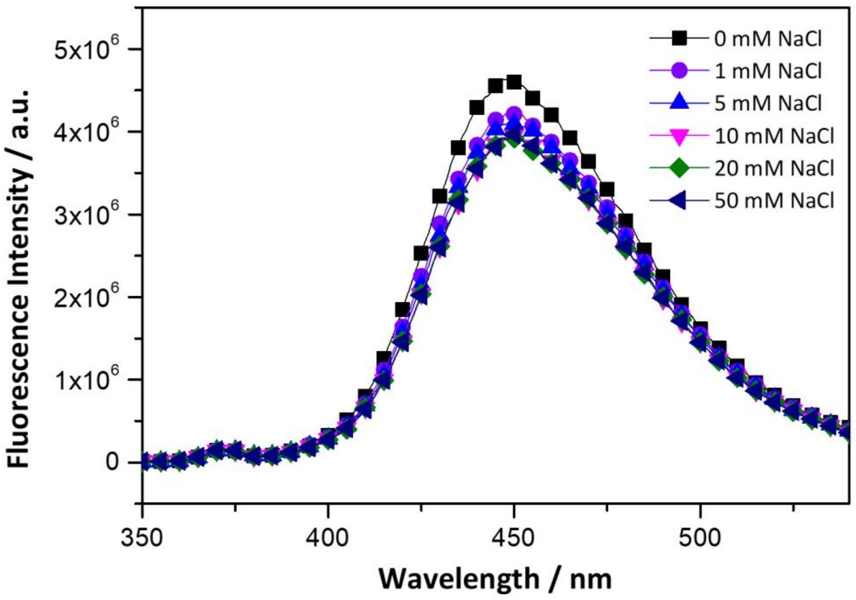

Figure S7: Fluorescence Emission Spectra of MOF Upon Titration with Increasing Concentrations of NaCl.

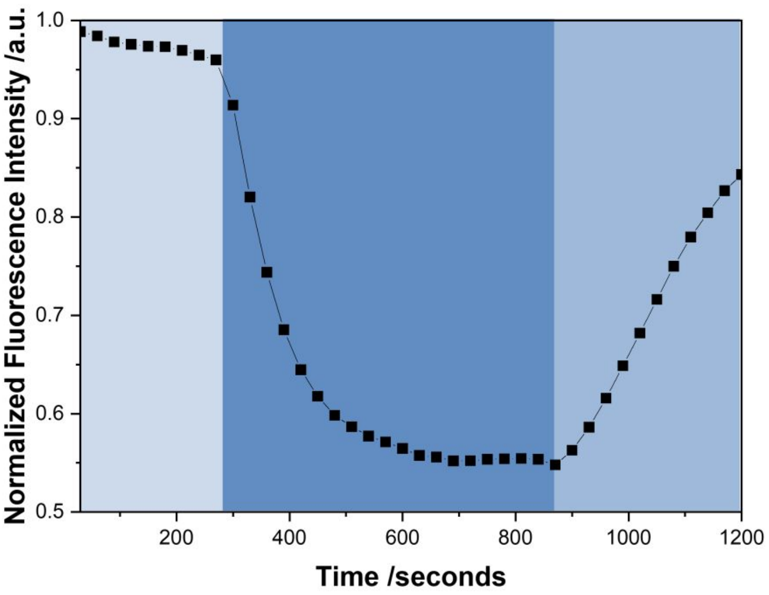

Figure S8: Fluorescence intensity time trajectory of a 0.125 mg/ml UiO-66-NH_2 MOF in deionized water. The trajectory was recorded at an emission wavelength 450 nm upon excitation

at 330 nm. The graph shows a pronounced initial decrease in normalized fluorescence intensity upon heating the sample to 70°C, stabilizing shortly thereafter, followed by a significant recovery in intensity after around 800 seconds when the temperature is returned to 20°C .

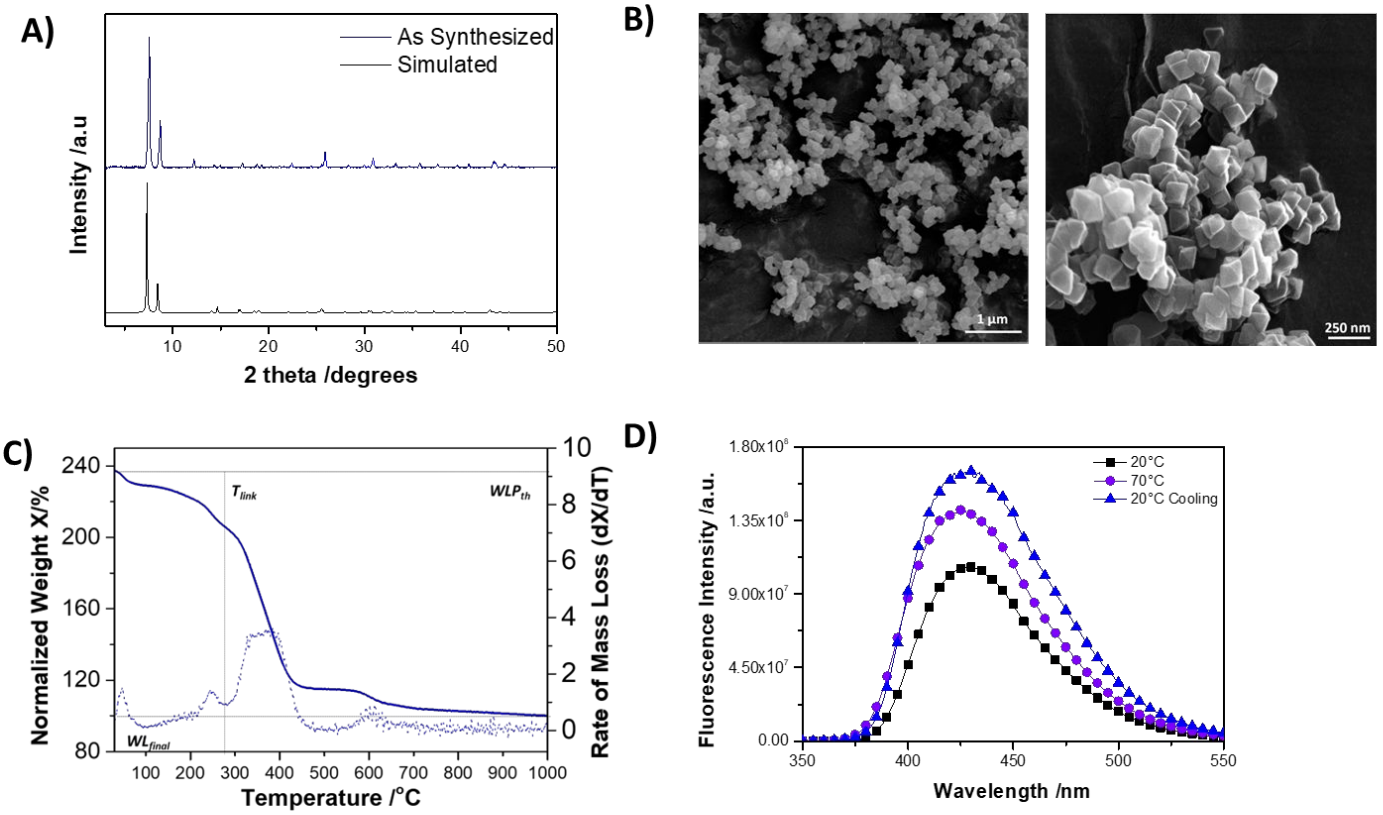

Figure S9: PXRD (A), SEM(B), TGA(C), and Emission Spectra of 0.125 mg/mL UiO-66-NH_2 prepared in the absence of modulator in 10 mM HEPES and 150 mM NaCl at 20 °C, 70 °C, and after cooling back to 20° (D).

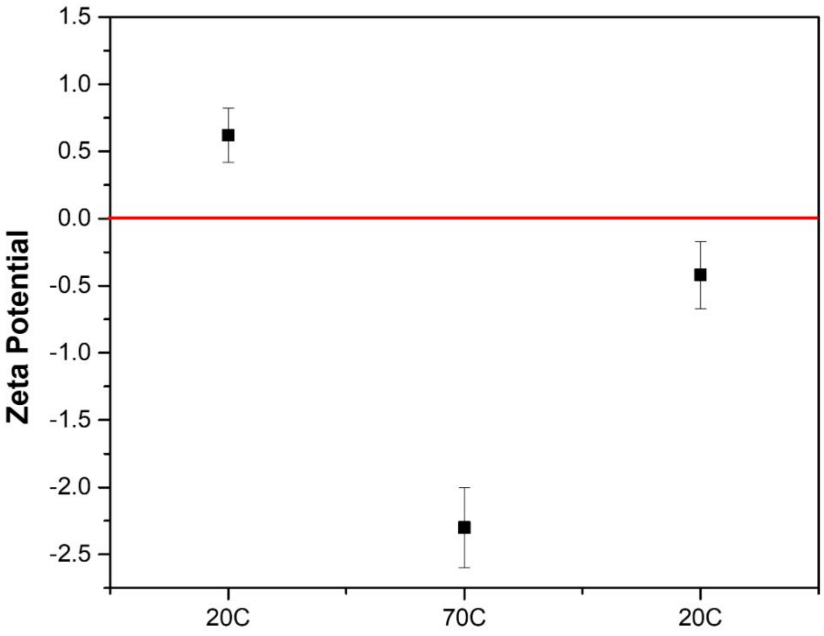

Figure S10: Zeta potential measurements of UiO-66-NH_2 suspension at different temperatures. The graph illustrates the surface charge at 20°C, 70°C, and at 20°C after cooling. There is a notable decrease in zeta potential with increasing temperature, suggesting increased particle stability at higher temperatures. Upon cooling, the zeta potential does not return to the original value, indicating a possible permanent change in surface characteristics or particle size distribution.

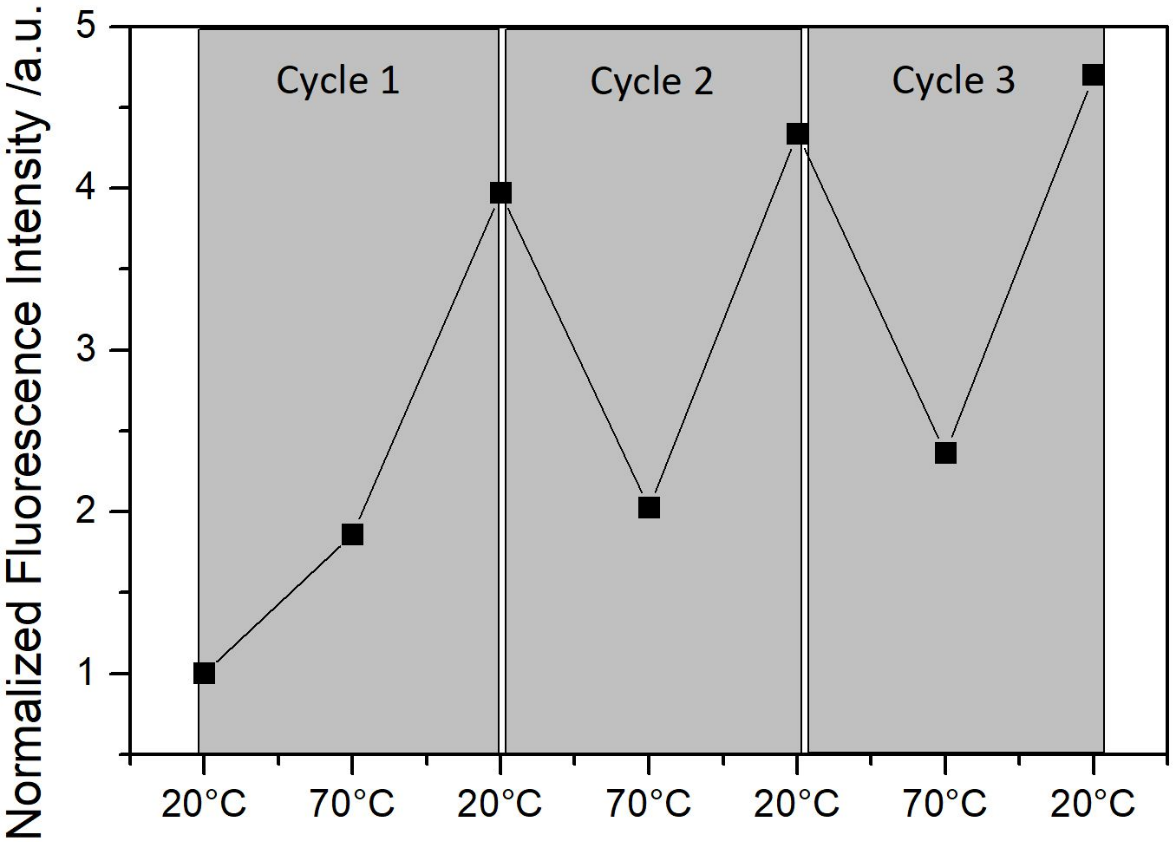

Figure S11: Normalized fluorescence intensity as a function of temperature during three thermal cycles.

.
